# Attention-Deficit Hyperactive Disorder among Primary School Children in Menoufia Governorate, Egypt

**DOI:** 10.1155/2014/257369

**Published:** 2014-12-10

**Authors:** Taghreed Farahat, Mohammad Alkot, Afaf Rajab, Reda Anbar

**Affiliations:** ^1^Family Medicine Department, Menoufia University, Menoufia, Egypt; ^2^Psychiatry Department, Menoufia University, Menoufia, Egypt

## Abstract

*Background*. Attention-deficit hyperactivity disorder (ADHD) is the most commonly diagnosed behavioral problem in children. Global variations in diagnostic criteria and rating scales of ADHD either by DSM-IV or ICD 10 may contribute to variations in its prevalence. *Objectives*. The study was conducted to estimate the prevalence of ADHD and to determine its risk factors. *Methodology*. A cross-section comparative study was conducted in a randomly selected four primary schools in Menoufia governorate, Egypt. All children after a valid consent of their parents (*N*. 1362) were subjected to complete history taking, medical and psychological assessment, and IQ estimation. Their parents and teachers were subjected to the corresponding Arabic forms of Conner's questionnaire. Suspected cases were confirmed and categorized by DSM-IV criteria. The sample was divided into cases and controls to study the risk factors. *Results*. Prevalence of ADHD was 6.9% and the male : female ratio was 3.5 : 1. The main risk factors were neonatal problems (OR = 4.3), family history of psychiatric and medical illnesses (OR = 3.5 and 2.8), and male gender (OR = 2.9). *Conclusion*. Prevalence of ADHD among Menoufia school children was 6.9%. Dealing with its risk factors is mandatory for prevention, early management, and better outcome.

## 1. Introduction

Attention-deficit hyperactivity disorder (ADHD) is a neurobehavioral disorder that typically begins in childhood and often persists into adulthood. ADHD is characterized by developmentally inappropriate levels of attention and hyperactivity resulting in functional impairment in academic, family, and social settings [1]. The DSM-IV distinguishes between its three subtypes: mainly inattentive, mainly hyperactive-impulsive and combined subtypes [1]. ADHD is a clinically heterogeneous condition, in which symptoms overlap or cooccur with other conditions. ADHD is most often identified when children first start school [2]. However, its symptoms may persist and continue to cause impairment throughout adolescence and adulthood [2]. ADHD may also be identified in children of preschool age [3]. The worldwide prevalence of ADHD ranges from 5.29% to 7.1% (*5.4–8.7% in* Africa,* 6.24% in* Jordan,* 16.4%* in Saudi Arabia) [2, 4]. The etiology of ADHD is not clearly understood; there is a growing body of evidence for the involvement of genetic [5, 6] neurobiological [7] and environmental factors [8]. A range of environmental factors have been implicated in the etiology of ADHD including risk factors during pregnancy or early childhood, social risk factors, and gene-environment interactions [9, 10].

Rational and aim of the study: there is a lack of studies in Africa, Middle East, Arab world, and Egypt regarding the prevalence and risk factors of ADHD. This work was conducted to determine the prevalence of ADHD and its risk factors among primary school children in Menoufia governorate, Egypt.

## 2. Patients and Methods

### 2.1. Ethical Issues

This study was conducted after its approval by the ethical committee of the faculty of medicine, Menoufia University, Egypt. All participants gave a valid written informed consent after clear explanation of the study aims and techniques.

### 2.2. Type of the Study

A cross-section comparative study.

### 2.3. Site of the Study

The study was conducted in four primary schools: two in Menouf city and the others in Monshaat Sultan village, Menouf district. All were selected by a multistage stratified random sampling technique representing both urban and rural communities of Menoufia governorate, Egypt.

### 2.4. Study Investigators

1. Family medicine consultants did complete the medical assessments including full history taking and data collection from parents and teachers.
2. Psychologist performed the IQ testing and explained study tools to both parents and teachers.
3. Psychiatric consultant completed the psychiatric assessment and confirmed the diagnosis.

### 2.5. Target Population

Primary school children in Menoufia governorate, Egypt, had the same chance to be randomly selected in the study sample.

### 2.6. Population of the Study

All school children (*N*. 1362) in the randomly selected four schools representing both urban and rural communities in Menoufia governorate, Egypt. 

*Exclusion Criteria.* They are as follows: children with IQ scores less than 80% (no. 18); a neurological handicap (no. 3), and chronic illness (no. 11).

### 2.7. Diagnostic Tools

Wechsler intelligence scale for children; Arabic forms of Conner's questionnaires for both parent and teacher; and the DSM-IV criteria for diagnosis and grading of ADHD [11] were validated and widely used in Egypt.

### 2.8. Procedure of the Study

The randomly selected children (*N*. 1362) in presence of their parents were subjected to complete history taking, comprehensive clinical examinations, and psychological assessment by a psychiatrist and IQ testing by a psychologist. Parents and teachers were invited after their consent to fill the corresponding form of Arabic Conner's questionnaire. They were instructed to rate the child's behavior at home and at school during the past six months.

### 2.9. Selection of Cases

Those cases were confirmed to have ADHD by psychological examination using DSM IV criteria after being positive in the medical and psychological examination as well as Conner's questionnaire.

### 2.10. Selection of Controls

Children gave negative results regarding diagnosis of ADHD. They were matched for age and sex to the patient group from the same schools and the same classes (see Table 1).

### 2.11. Statistical Analysis

Collected data were statistically analyzed utilizing the Statistical Package for the Social Sciences (SPSS) software program version 18, using a personal computer. Qualitative data were expressed as number and percent and tested by the chi-squared test. However the quantitative data were expressed as mean and standard deviation and tested by the *t*-test. Crude and adjusted odds ratio using logistic regression analysis were calculated at 95% confidence intervals. Stepwise backward binomial ordinary logistic regression model was used in this analysis where the dependent variable is a dichotomous categorical variable (ADHD) and the independent variables are age, sex, neonatal problems, and so forth. The purpose was to analyze the effects of the independent variables on the dependent one. All the significant independent variables tested by chi-squared test (*P*  value < 0.05) and crude odds ratios (>1 and their confidence intervals not including 1) were entered in the regression model analysis.

## 3. Results

The total prevalence of ADHD among basic school children was 6.9% (6.8% in urban and 6.9% in rural). The mean age of school children having ADHD ± SD was 8.25 ± 1.62. There was a higher prevalence of ADHD in male than female children with male to female ratio of nearly 3.5 : 1. There was no statistically significant difference regarding most demographic data as regards age, mean age of parents, birth order, and residency. The highest prevalence of ADHD was among seven-year-old school children (22.1%). The younger the birth order, the higher the prevalence of ADHD. Males had a higher prevalence than females (10.9% versus 3.5%). The proportion of inattentive type was 31.5% and was slightly higher in females (1 : 1.9), combined type was 48.0% of the total cases and was predominant in males (2.1 : 1). However, the hyperactive-impulsive type represented 20.5% with a male to female ratio of 1.4 : 1. The major risk factors for ADHD using logistic regression analysis were neonatal problems (cyanosis, low birth weight, jaundice, and incubation for other causes), family history of psychiatric illness, gender, family history of medical illness,* consanguinity*, antenatal illness and drug use, and family size greater than four. An unemployed mother in this study was a protective factor, not a risk factor. Other significant risk factors did not reach a statistically significant level after adjusting the odds ratio by logistic regression analysis. These were low maternal education, paternal smoking, an unemployed father, low paternal education, delivery by caesarian section, and disrupted families.

*New Results of This Work*

1. An unemployed mother is a protective factor and not a risk factor.
2. Low parental education is not risky for ADHD.
3. Parental use of smoking tobacco is not risky for ADHD.
4. Disrupted families and delivery type (caesarian section) are not risk factors for ADHD.

## 4. Discussion

The worldwide prevalence of ADHD among school children is 5–7% [1]. In the current study (Table 2), the prevalence of ADHD based on the DSM-IV criteria was found to be 6.9% among primary school children of Menoufia governorate, Egypt. which is higher than the results of another study (5.1%) done in the same governorate in 2007 by Elwan et al. [12]. Also it was higher than the results of another study (6%) done in Assuit city, Egypt [13]. However our results were lower than results in Suez Canal University Hospital by Magda et al., which found the prevalence to be 13.6%. This latter difference may be attributed to a hospital based sample versus a community based one in this study [14].

The current study (Tables 4 and 5) revealed that neonatal health problems such as low birth weight, cyanosis, neonatal jaundice, and incubation for other causes had about four and half folds increased chance to lead to ADHD (adjusted OR = 4.3). This result was in concordance with the current evidence that prevalence of ADHD increases with hypoxia and prematurity [15]. A significant excess of psychiatric morbidity compared with controls were found in low birth weight children [16], which appear to be associated with a greater risk for symptoms of inattention than hyperactivity/impulsivity subtypes [17].

The current study revealed that children having a positive family history of psychiatric and medical illnesses had 3.6 and 2.85 folds increased chance to get ADHD (adjusted OR = 3.6 and 2.85, resp.). The current opinion is that ADHD is a mixture of genetics and environmental factors. However the pathophysiology is unclear at this time, although results from various types of neuroimaging techniques suggest there are differences between individuals with and without ADHD in the brain, such as thinner regions of the cortex [18, 19].

The current study (Table 3) revealed that males had a higher prevalence of ADHD than females (3.5 : 1) and had about three folds increased chance of having ADHD (adjusted OR = 2.9). This was in concordance with several studies that showed a well-documented gender difference in the prevalence of ADHD.

The study revealed (Tables 4 and 5) that children growing up in large families of more than four had about one and half folds increased chance to get ADHD (adjusted OR: 1.5) which was in concordance with a study done in Dammam, KSA, which reported that the larger the family size, the higher the prevalence of ADHD. Large family size accounts for various psychological problems. They may be among the contributing factors to ADHD due to impact on interpersonal relationships between family members [20], bearing in mind that the family's communicative culture and social factors have a significant role and function in the formation of inter- and intrapersonal relations. There is a need for further cross-cultural studies to investigate the relationship between family problems and their contributions to psychological disorders and ADHD [21].

The current study (Tables 4 and 5) demonstrated that consanguinity had about one and half folds increased chance to lead to ADHD (adjusted OR = 1.4) which was in concordance with Jordanian study which found that the prevalence of ADHD was 34.8% among consanguineous families and the inattentive subtype was more common than others. Another study in Qatar showed a significant relationship between ADHD symptoms and consanguineous parents [22].

The study (Tables 4 and 5) showed that both antenatal illness and drugs had about one and half folds increased risk for ADHD (adjusted OR = 1.47), which was in concordance with Nigg and Breslau, who reported that maternal drug abuse and cigarette smoking during pregnancy may lead to the development of ADHD in children [23].

Chronic exposure to smoking in pregnancy has been extensively studied, where an evidence of a dose-response relationship between the numbers of cigarettes smoked and ADHD severity was confirmed. Smoking in pregnancy is known to result in lower birth weight. Also exposure to carbon monoxide or altered placental function could account for development of ADHD [23]. In the current work (Tables 4 and 5) paternal smoking was associated as a risk factor for ADHD but did not reach a statistically significant level, while maternal smoking was not associated with ADHD. Paternal and maternal smoking results of this current study contradicted other studies in several countries. This difference may be attributed to ethical and cultural aspects in Egypt as female tobacco smoking, drug, and alcohol use are not common. Maternal tobacco smoking is known as one of the major risks for developing ADHD in their offspring [23].

In the current study (Tables 4 and 5), disrupted family environment was not a risk factor for ADHD (*P*  value < 0.05, crude OR was 1.6 and adjusted OR = 1.1). This was in comparison to a study in Saudi Arabia which reported the prevalence of hyperactivity-impulsivity disorder was found to be significantly higher in boys living with single parents [24]. Parental separation and divorce has been shown to have negative impacts on child behavior, such as inconsistent parenting, more punishment, violence, and criticism [25].

Parental occupation and education level are known to affect the prevalence of ADHD. The current study (Tables 4 and 5) demonstrated that an unemployed mother was protective of having a child with ADHD whereas the unskilled paternal occupation and low educational levels of mother and father were not risk factors for ADHD. These results differed from a Swedish study which found a strong association between the prevalence of ADHD and socioeconomic level. Many authors showed limited maternal education, single-parent families, and reduced family welfare were associated with children having ADHD [26].

Many familial factors may influence ADHD, for example, low parental education, stress factors, and a greater risk of childhood adversity. Family environment may influence development of ADHD and related behavioral problems. Childhood neglect, posttraumatic stress disorder, abuse, and institutionalization have a negative effect on brain development [26].

## 5. Conclusion

Prevalence of ADHD among school children in Menoufia governorate, Egypt, was 6.9%. ADHD is associated with many risk factors either modifiable or nonmodifiable such as* consanguinity*, antenatal illness, antenatal drug use, abnormality at birth, large family size, family history of psychiatric or medical illness, and sex. Prevention, early detection, and management of its modifiable risk factors should be undertaken alongside increasing community awareness. 

*Limitations of the Study*

1. Difficulty in recalling the history of antenatal periods and drug names from some illiterate mothers that is overcome by recalling of data from their family folders.
2. Low level of community awareness regarding childhood behavioral problems.
3. Communal misperception of psychiatric conditions.

## Figures and Tables

**Table 1:** 

	Definition	Specificity of definitions
1	Family history of psychiatric illness	ADHD, autism, depression, anxiety, mania, and/or associated behavioral disorders
2	Family history of medical illness	DM, hypertension, hepatic, renal, and any chronic illnesses
3	**Consanguinity**	2nd degree relatives
4	Antenatal illness and drugs	Major and/or chronic antenatal diseases as gestational diabetes, hypertension, preeclampsia, and so forth
5	**There **was **association** but **statistically** did not **reach a** significant level	The crude odds ratio was significant while the adjusted **OR** became **un**significant

**Table 2:** Demographic data of the studied group.

Variable	ADHD		NO ADHD		*X* ^2^	*P* value	Odds ratio at 95% CI
	No	%	No	%			
Age in years							
6-	18	18.78	229	18.1	3.06	0.690
7-	21	22.33	221	17.4		
8-	17	10.08	211	16.6		
9-	15	15.95	206	16.3		
10-	13	13.82	202	15.9		
11-12	10	10.62	199	15.7		
Gender							
Male	73	77.6	594	46.8	33.2	0.0001	3.94
Female	21	22.4	674	53.2			
Birth order							
Eldest	25	26.6	393	31	2.94	0.229
Youngest	31	33	471	37.1		
Others	38	40.4	404	31.9		
Residency							
Rural	45	47.9	615	48.5	0.01	0.90
Urban	49	52.1	653	51.5		
School type							
Governmental	61	64.9	863	75.9	0.4	0.52
Private school	33	35.1	405	24.1		

**Table 3:** Gender distribution of ADHD subtypes.

Variant	Male (*N*. 73)		Female (*N*. 21)		*X* ^2^	*P* value
	*N*.	%	*N*.	%		
ADHD C	35	48.0	5	23.8	7.80	<0.05
ADHD H	15	20.5	3	14.3		
ADHD I	23	31.5	13	61.9		

**Table 4:** Risk factors for ADHD.

Variable	ADHD *N*. 94		Control *N*. 1268		*X* ^2^	*P* value	Crude OR (95% confidence interval)
	No	%	No	%			
(Mean ± SD)	8.25 ± 1.62		8.57 ± 1.78		*t*-test 1.73	0.07	
Neonatal problems							
Yes	45	47.9	162	12.8	83.6	<0.05	6.2 (4.0–9.7)
No	49	52.1	1106	87.2			
FH of psychiatric illness							
Yes	62	66	390	30.8	48.9	<0.05	4.4 (2.8–6.8)
No	32	34	878	69.2			
Sex							
Male	73	77.7	594	46.8	33.2	<0.05	3.94 (2.4–6.49)
Female	21	22.3	674	53.2			
FH of medical illness							
Yes	59	62.8	397	31.3	47.9	<0.05	3.69 (2.4–5.7)
No	35	37.2	871	68.7			
Family size							
>4	68	72.3	727	57.3	6.3	<0.05	1.95 (1.2–3.1)
<4	26	27.7	541	42.7			
Consanguinity							
Yes	56	59.6	572	45.1	7.36	<0.05	1.8 (1.8–2.7)
No	38	40.4	696	54.9			
Antenatal illness & drugs							
Yes	62	66	669	52.8	6.1	<0.05	1.73 (1.1–2.7)
No	32	34	599	47.2			
Paternal smoke tobacco							
Yes	59	62.8	643	50.7	4.9	<0.05	1.63 (1.1–2.5)
No	35	37.2	625	49.3			
Family structure							
Disrupted	35	37.2	340	26.8	4.76	<0.05	1.6 (1.1–2.5)
Preserved	59	62.8	928	73.2			
Delivery type							
Caesarian	55	58.5	592	46.7	4.91	<0.05	1.6 (1.1–2.5)
Normal	39	41.5	676	53.3			
Paternal education							
Illiterate & primary	49	52.1	510	40.2	5.13	<0.05	1.6 (1.1–2.5)
Secondary & high	45	47.9	758	59.8			
Paternal work							
Employed	52	55.3	562	44.3	4.27	<0.05	1.6 (1.02–2.4)
Unemployed	42	44.7	706	55.7			
Paternal education							
Illiterate & primary	52	55.3	544	42.9	5.48	<0.05	1.6 (1.1–2.5)
Secondary & high	42	44.7	724	57.1			
Maternal smoking tobacco							
Yes	14	14.9	168	13.2	0.2	0.6	1.14 (0.6–1.9)
No	80	85.1	1100	86.8			
Maternal occupation							
Employed	38	40.4	654	51.6	4.35	0.04	0.6371 (0.4–0.97)
Unemployed	56	59.6	614	48.4			

**Table 5:** Logistic regression analysis of studied significant risk factors.

Variable	Adjusted odds ratio	95% confidence interval
Neonatal problems	4.3	3.3–7.6^**^
FH of psychiatric illness	3.59	1.98–5.89^**^
Sex	2.92	2.1–5.52^**^
FH of medical illness	2.85	1.99–5.98^**^
Consanguinity	1.4	1.22–1.95^**^
Antenatal illness and drug use	1.47	1.1–1.95^**^
Family size > 4	1.51	1.10–2.3^**^
Low maternal education	1.4	0.91–1.4^*^
Paternal smoking	1.3	0.9–1.3^*^
Unemployed father	1.3	0.9–1.5^*^
Low paternal education	1.2	0.88–1.3^*^
Delivery type—caesarian section	1.2	0.89–1.4^*^
Disrupted families	1.1	0.87–1.3^*^

^**^Significant, ^*^nonsignificant.
